# Clinical Characteristics and Inflammatory Immune Responses in COVID-19 Patients With Hypertension: A Retrospective Study

**DOI:** 10.3389/fphar.2021.721769

**Published:** 2021-10-25

**Authors:** Chaoran Wu, Guangbo Qu, Lei Wang, Shiyu Cao, Dandan Xia, Baolong Wang, Xiaoyun Fan, Changhui Wang

**Affiliations:** ^1^ Department of Cardiology, the First Affiliated Hospital of Anhui Medical University, Hefei, China; ^2^ Department of Epidemiology and Health Statistics, School of Public Health, Anhui Medical University, Hefei, China; ^3^ Department of Geriatric Respiratory and Critical Care, the First Affiliated Hospital of Anhui Medical University, Hefei, China

**Keywords:** inflammatory immune responses, hypertension, RAAS blockers, COVID-19, myocardial injury

## Abstract

Coronavirus disease (COVID-19) patients with cardiovascular and metabolic disorders have been found to have a high risk of developing severe conditions with high mortality, further affecting the prognosis of COVID-19. However, the effect of hypertension and angiotensin-converting enzyme inhibitors (ACEI) and angiotensin receptor blocker (ARB) agents on the clinical characteristics and inflammatory immune responses in COVID-19 patients is still undefined. In this study, 90 COVID-19 patients were divided into hypertension and nonhypertension groups. The hypertension group was divided into well-controlled and poorly controlled subgroups based on blood pressure levels; moreover, hypertensive patients were also divided into ACEI/ARB and non-ACEI/ARB subgroups according to the administration of ACEI/ARB antihypertensive agents. The clinical characteristics of and inflammatory immune biomarker levels in the different groups of COVID-19 patients were compared, and the association between the combined effect of hypertension with ACEI/ARB antihypertensive agents and the severity of COVID-19 was examined. The results showed that the levels of aminotransferase (AST) and hs-cTnI were higher in the hypertension group compared with the nonhypertension group. The long-term use of ACEI/ARB agents in patients had statistically significantly lower AST, low-density lipoprotein cholesterol (LDL-C), and oxygen uptake and lower white cell count, neutrophil count, and levels of CD4, CD8, CRP, and PCT but without statistical significance. In addition, compared with COVID-19 patients without hypertension, hypertensive patients without the use of ACEI/ARB had a higher risk of developing severity of COVID-19 (for poorly controlled patients: OR = 3.97, 95% CI = 1.03–15.30; for well-controlled patients: OR = 6.48, 95% CI = 1.77–23.81). Hypertension could cause organ damage in COVID-19 patients, but the long-term use of ACEI/ARB agents may be beneficial to alleviate this injury.

## Introduction

**FIGURE 1:**
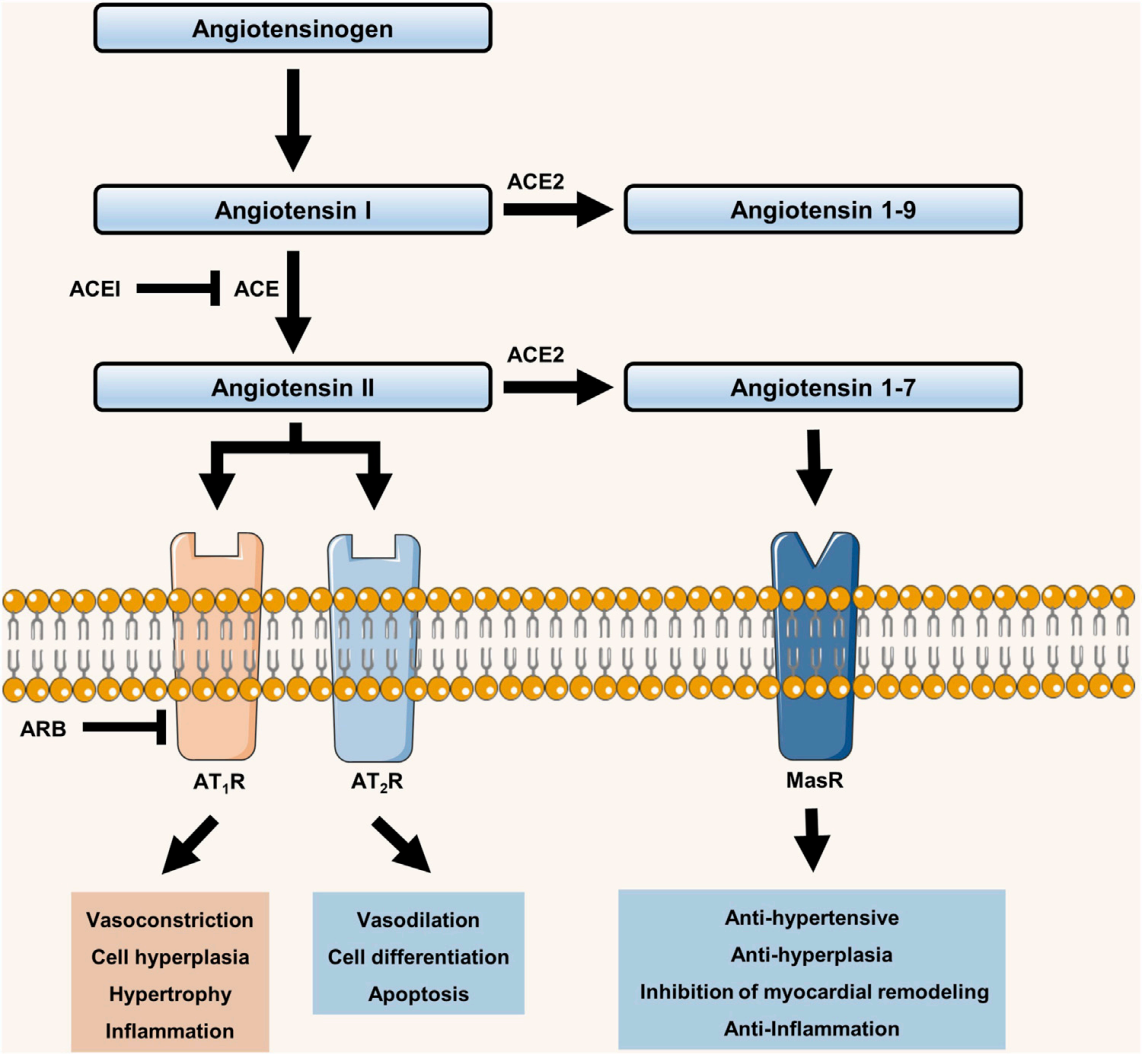
Schematic diagram of the renin–angiotensin–aldosterone system (RAAS).

Coronavirus disease (COVID-19) is caused by severe acute respiratory syndrome coronavirus 2 (SARS-CoV-2). It has developed into a high-risk pandemic that has affected the global population. By July 18, 2021, more than 190,000,000 COVID-19 patients have been confirmed worldwide (WHO, 2021). COVID-19 patients with cardiovascular conditions, especially hypertension, have been reported to be at a high risk of developing severe conditions and mortality, in turn affecting the prognosis of COVID-19 (Chen et al., 2020; Zhou et al., 2020; Li et al., 2020; Leung, 2020). Our previous study also provided evidence to confirm this (Xie et al., 2020). Among the well-known pathophysiological elements leading to essential hypertension, the renin–angiotensin–aldosterone system (RAAS) has a vital role (Lu et al., 2015). Many studies have also demonstrated that angiotensin (Ang) II and other components of the RAAS, such as Ang-(1–7) and aldosterone, contribute to the inflammatory response, which suggests that the activation or blockage of the RAAS is related to the inflammatory response in patients with COVID-19 (the RAAS system is presented in Figure 1) (Di Raimondo et al., 2012; El-Hashim et al., 2012). In addition, the dysregulation of immune response in patients with COVID-19 has been demonstrated (Leisman et al., 2020). Therefore, hypertension may contribute to the progression of COVID-19. Moreover, the angiotensin-converting enzyme 2 receptor (ACE2 receptor) in the RAAS is considered to play an important role. As a monocarboxypeptidase, it converts the vasoconstrictor Ang II to Ang-(1–7) and exerts a variety of organ-protective properties (Danser et al., 2020; Serfozo et al., 2020). However, the ACE2 receptor has also been reported to be a functional receptor for coronavirus-induced infection through binding of the spike protein of SARS-CoV-2 and ACE2 (Bosso et al., 2020). Current research has shown that an increase in the ACE2 receptor aids COVID-19 infection (Esler and Esler, 2020). In addition, ACE inhibitors (ACEIs) and angiotensin receptor blockers (ARBs) are widely used as antihypertensive drugs, which can enhance ACE2 expression (Gottlieb et al., 1993; Bauer et al., 1995). However, there is insufficient clinical evidence regarding the negative effects of the long-term use of these drugs in COVID-19 patients. Therefore, arbitrarily adjusting the use of antihypertensive drugs is not recommended (Esler and Esler, 2020)

In this study, we described the clinical characteristics, inflammatory markers, and outcomes in COVID-19 patients with hypertension. We also preliminarily explored the effect of ACEI/ARB agents on the clinical characteristics of COVID-19. This study provided additional evidence for the beneficial effects of ACEI/ARB agents on COVID-19 in patients with hypertension.

## Methods

### Participants

This study enrolled 90 patients with confirmed COVID-19 infection from the Z6 and Z11 infectious departments of the Union Hospital Tongji Medical College of Huazhong University of Science and Technology. Hospital admission varied from February 15, 2020, to February 28, 2020, and information on all cases was recorded on March 14, 2020.COVID-19 was diagnosed based on the Diagnosis and Treatment Protocol for Novel Coronavirus Pneumonia released by the National Health Commission of the PRC (Wei, 2020). Blood pressure was monitored and measured during hospitalization, with the patient seated. Hypertension was diagnosed either on the basis of blood pressure measurements (systolic blood pressure ≥ 140 mmHg and/or diastolic blood pressure ≥ 90 mmHg) at least twice during the first and second days after hospital admission or on the basis of whether the patient was undergoing hypotensive therapy. Of the enrolled patients, 31 (34.4%) had a confirmed diagnosis of hypertension. According to the current guidelines (Williams et al., 2018), well-controlled hypertension was defined as a blood pressure < 140/90 mmHg during the first and second days after hospital admission in COVID-19 patients with hypertension. Therefore, of the 31 COVID-19 patients with hypertension, 17 were classified as having well-controlled hypertension and 14 were classified as having poorly controlled hypertension. In addition, according to the antihypertensive history before hospital admission, we further divided the hypertension cases into ACEI/ARB (n = 6) and non-ACEI/ARB (n = 25) groups.

### Laboratory Confirmation of COVID-19

To diagnose COVID-19, throat swab samples obtained from all patients were subjected to real-time reverse transcriptase–polymerase chain reaction at least twice according to the protocol described previously (Huang et al., 2020). The test was performed by the Union Hospital Tongji Medical College of Huazhong University of Science and Technology.

### Classification of COVID-19

The clinical classification of the severity of COVID-19 was based on the Diagnosis and Treatment Protocol for Novel Coronavirus Pneumonia released by the National Health Commission of the PRC (Huang et al., 2020). Mild COVID-19 was defined as patients with mild symptoms and no imaging abnormalities. Moderate COVID-19 was defined as patients with respiratory infection symptoms, such as fever, cough, and pneumonia manifestation on imaging. Severe COVID-19 was defined as patients with a respiratory rate of ≥30 breaths/min, a resting fingertip oxygen saturation of ≤ 93%, or an oxygen partial pressure (PaO2)/fraction of inspired O_2_ (FiO_2_) of ≤ 300 mmHg (1 mmHg = 0.133 kPa). Critical COVID-19 was defined as patients with respiratory failure requiring mechanical ventilation, symptoms of shock, or multiple organ dysfunction requiring intensive care. In our study, mild and moderate cases were categorized as the nonsevere group and severe and critical cases were categorized as the severe group.

### Data Collection

All data were collected from electronic medical records. Epidemic data included age, sex, signs and symptoms, comorbidities, and medication history. Laboratory findings included routine blood tests, arterial blood gas, cardiac markers, renal and liver function, blood lipid levels, and immune indicators. Computed tomography on admission was performed for radiological assessment. Oxygen inhalation and the use of antibiotics were recorded.

### Statistical Analysis

The data analysis was performed using SPSS software version 24.0. Continuous variables with a nonnormal distribution are presented as median (M) and interquartile range (IQR). Discontinuous variables are described as numbers (n) and percentages. Clinical characteristics and inflammatory immune biomarker levels were first compared between COVID-19 patients with different conditions of hypertension. In addition, these comparisons were also performed between COVID-19 patients with different ACEI/ARB use conditions. All comparisons were conducted using nonparametric tests or Fisher’s exact probability tests. All significance levels were further adjusted using the Bonferroni correction for pairwise comparisons among the three groups. Univariate logistic regressions were performed to preliminarily determine the factors associated with the severity of COVID-19. Multivariate logistic regression was conducted to examine the association between the blood pressure control, ACEI/ARB use, and the severity of COVID-19 adjusted according to age and sex. All statistical tests were two-sided, and *p*-values less than 0.05 indicated statistical significance.

## Results

### Demographic and Clinical Characteristics of Patients

The median age of the 90 patients was 64.00 years (IQR: 55.50–73.00). Of the included cases, 24 (26.7%) were severe cases. There were 31 (34.4%) patients with hypertension, of which 16 (51.7%) were women and 15 (48.4%) were severe cases. The most documented comorbidities in COVID-19 patients with hypertension were diabetes (32.3%), followed by coronary heart disease (29.0%), heart failure (6.5%), respiratory disease (6.5%), cancer (6.5%), and atrial fibrillation (3.2%). Compared with nonhypertensive patients, the prevalence of diabetes (32.3 vs 11.9%, *p* = 0.025) and coronary heart disease (29.0 vs 3.4%, *p* = 0.003) was significantly higher. The most common symptoms of the onset were fever (74.4%) and cough (47.8%) in all COVID-19 patients. In the subgroup of patients with hypertension, 71.0% had fever and 48.4% had cough (Table 1).

**TABLE 1:** The comparison of demographic and clinical characteristics, laboratory and radiology findings, clinical measures, clinical outcomes, and severity of COVID-19 among well-controlled, poorly controlled, and nonhypertension groups.

	All patients (n = 90)	Hypertension (n = 31)		Nonhypertension (n = 59)	*P* ^a^	*P* ^b^
		Well-controlled (n = 17)	Poorly controlled (n = 14)			
*Demographic characteristics*						
Sex (n, %)^c^						
Female	49 (54.4)	8 (47.1)	8 (57.1)	33 (55.9)	0.824	0.818
Age (years) [M (Q1, Q3)]^d^	64.00 (55.50, 73.00)	65.00 (53.00, 73.00)	62.5 (55.50, 73.75)	64.00 (54.00, 72.00)	0.714	0.967
*Clinical characteristics*						
Clinical classification (n, %)^c^					0.001	0.002
Severe	24 (26.7)	9 (52.9)	6 (42.9)	9 (15.3)		
Nonsevere	66 (73.3)	8 (47.1)	8 (57.1)	50 (84.7)		
*Preadmission comorbidities (n, %)* ^c^						
CHD	10 (11.1)	6 (35.3)	2 (14.3)	2 (3.4)	0.003	0.001
HF	2 (2.2)	0 (0)	2 (14.3)	0 (0)	0.116	0.023
AF	1 (1.1)	0 (0)	1 (7.1)	0 (0)	0.344	0.156
Diabetes	17 (18.9)	7 (41.2)	3 (21.4)	7 (11.9)	0.025	0.022
Respiratory disease	5 (5.6)	1 (5.9)	1 (7.1)	3 (5.1)	1.000	1.000
Cancer	8 (8.9)	2 (11.8)	0 (0)	6 (10.2)	0.710	0.639
*Signs and symptoms (n, %)* ^c^						
Fever	67 (74.4)	11 (64.7)	11 (78.6)	45 (76.3)	0.617	0.562
Cough	43 (47.8)	7 (41.2)	8 (57.1)	28 (47.5)	1.000	0.706
Sore throat	8 (8.9)	1 (5.9)	1 (7.1)	6 (10.2)	0.710	1.000
Expectoration	16 (17.8)	3 (17.6)	4 (28.6)	9 (15.3)	0.563	0.511
Shivering	10 (11.1)	1 (5.9)	1 (7.1)	8 (13.6)	0.484	0.789
Headache	5 (5.6)	1 (5.9)	0 (0)	4 (6.8)	0.656	1.000
Runny nose	4 (4.4)	1 (5.9)	0 (0)	3 (5.1)	1.000	1.000
Diarrhea	13 (14.4)	1 (5.9)	2 (14.3)	10 (16.9)	0.530	0.609
Muscular soreness	8 (8.9)	3 (17.6)	0 (0)	5 (8.5)	1.000	0.242
Feeble	18 (20.0)	3 (17.6)	2 (14.3)	13 (22.0)	0.588	0.927
Chest tightness	26 (28.9)	8 (47.1)	2 (14.3)	16 (27.1)	0.631	0.134
Anhelation	12 (13.3)	2 (7.1)	1 (7.1)	9 (15.3)	0.534	0.900
Temperature (°C) [M (Q1, Q3)]	38.05 (37.50, 38.78)	38.20 (37.00, 38.60)	38.00 (37.20, 38.80)	38.05 (37.50, 38.95)	0.467	0.823
*Average blood pressure during hospitalization, mmHg [M (Q1, Q3)]* ^d^						
Systolic blood pressure, mmHg	125.00 (116.00, 131.00)	129.00 (110.00, 132.25)	136.00 (131.50, 144.50)^e^	121.00 (113.25, 126.00)^f^	0.000	0.000
Diastolic blood pressure, mmHg	76.00 (72.00, 81.00)	78.50 (73.75, 82.00)	79.00 (71.50, 87.50)	75.50 (71.00, 79.00)	0.096	0.239
*Laboratory findings*						
Blood routine [M (Q1, Q3)]^d^						
White cell count, ×10^9^/L	5.04 (4.28, 5.95)	5.76 (4.50, 6.76)	4.95 (3.81, 6.61)	4.96 (4.24, 5.79)	0.073	0.058
Neutrophil count, ×10^9^/L	3.22 (2.51, 4.09)	3.55 (3.27, 4.12)	2.94 (1.32, 4.66)	3.07 (2.45, 4.07)	0.146	0.116
Lymphocyte count, ×10^9^/L	1.24 (0.90, 1.56)	1.51 (0.99, 1.77)	1.15 (0.99, 1.80)	1.22 (0.84, 1.50)	0.165	0.235
Hemoglobin, g/L	118.00 (112.00, 128.25)	118.00 (113.50, 131.00)	118.00 (113.00, 127.75)	119.00 (111.00, 126.00)	0.575	0.848
Platelet count, ×10^9^/L	214.00 (182.50, 291.75)	223.00 (197.50, 295.50)	232.50 (171.25, 362.75)	208.00 (183.00, 288.00)	0.386	0.652
HCT (%)	34.85 (33.08, 37.55)	35.40 (32.50, 38.80)	35.15 (34.10, 40.00)	34.40 (33.00, 36.90)	0.136	0.311
*Cardiac markers [M (Q1, Q3)]* ^d^						
hs-cTnI (ng/L)	3.10 (1.48, 7.88)	4.20 (1.90, 4.20)	4.70 (1.68, 15.13)	2.60 (1.30, 5.60)	0.057	0.162
AST (U/L)	25.00 (19.00, 35.25)	28.00 (20.50, 43.00)	32.50 (20.75, 61.25)	23.00 (19.00, 31.00)	0.018	0.048
CK (U/L)	65.00 (51.50, 93.50)	59.00 (31.00, 117.50)	76.50 (67.25, 96.00)	64.00 (51.00, 88.50)	0.544	0.117
CK-MB (U/L)	0.70 (0.45, 2.50)	0.70 (0.30, 1.00)	1.00 (0.65, 12.00)	0.70 (0.40, 7.85)	0.651	0.190
LDH(U/L)	191.50 (154.00, 244.25)	196.00 (162.00, 225.00)	204.00 (171.75, 360.25)	183.00 (150.00, 243.00)	0.194	0.339
*Renal and liver function [M (Q1, Q3)]* ^d^						
Cr (μmol/L)	69.50 (63.75, 83.00)	79.00 (64.00, 97.00)	78.00 (63.50, 83.50)	68.90 (63.00, 76.00)	0.081	0.200
BUN (mmol/L)	4.55 (3.50, 5.44)	4.60 (4.10, 5.50)	4.90 (3.58, 6.73)	4.40 (3.40, 5.20)	0.210	0.427
ALT (U/L)	25.50 (17.00, 39.25)	33.18 (14, 65)	40.29 (12, 119)	28.69 (8, 100)	0.200	0.414
*Blood lipid level [M (Q1, Q3)]* ^d^						
TG (mmol/L)	1.31 (0.97, 1.90)	1.30 (0.81, 1.65)	1.56 (1.12, 2.29)	1.26 (0.98, 1.84)	0.606	0.419
LDL-C (mmol/L)	2.24 (1.87, 2.55)	2.30 (1.86, 2.58)	2.40 (1.79, 2.70)	2.22 (1.90, 2.48)	0.635	0.882
HDL-C (mmol/L)	1.21 (1.04, 1.50)	1.25 (1.11, 1.68)	1.22 (0.98, 1.52)	1.20 (1.02, 1.49)	0.526	0.535
*Lymphocyte subsets [M (Q1, Q3)]* ^d^						
CD4 (%)	43.80 (39.04, 51.51)	45.68 (35.42, 51.28)	48.05 (42.25, 58.78)	43.34 (37.15, 51.00)	0.452	0.260
CD8 (%)	23.55 (17.82, 29.99)	21.34 (17.58, 25.60)	23.79 (5.25, 41.39)	24.69 (9.80,39.56)	0.381	0.643
*Inflammatory markers [M (Q1, Q3)]* ^d^						
IL-6 (pg/ml)	9.78 (5.31, 27.81)	9.44 (5.12, 16.70)	16.93 (6.43, 27.87)	8.97 (5.00, 33.94)	0.830	0.688
CRP (mg/L)	3.78 (1.03, 3.78)	3.41 (0.50, 26.92)	10.70 (2.40, 44.95)	2.95 (0.78, 23.98)	0.355	0.319
PCT (ng/L)	0.07 (0.05, 0.16)	0.07 (0.04, 0.20)	0.14 (0.07,0.21)	0.07 (0.04, 0.16)	0.208	0.327
*Coagulation test [M (Q1, Q3)]* ^d^						
APTT (sec)	36.85 (34.43, 39.80)	37.20 (35.90, 39.50)	38.70 (35.63, 42.40)	35.90 (33.70, 39.30)	0.033	0.088
PT (sec)	13.50 (12.90, 14.00)	13.30 (12.75, 13.75)	13.35 (12.78, 14.30)	13.60 (13.00, 14.10)	0.278	0.310
D-dimer (mg/L)	0.50 (0.30, 1.30)	0.60 (0.40, 1.53)	0.98 (0.38, 1.35)	0.47 (0.20, 1.14)	0.144	0.328
*Arterial blood gas [M (Q1, Q3)]* ^d^						
PH	7.41 (7.40, 7.43)	7.41 (7.38, 7.43)	7.41 (7.38, 7.46)	7.41 (7.40, 7.43)	0.682	0.682
Lactic acid (mmol/L)	1.90 (1.50, 2.20)	2.05 (1.73, 2.15)	2.20 (1.80, 2.70)	1.80 (1.28, 2.08)	0.048	0.110
*Radiology findings*						
Abnormalities on chest CT (n, %)^c^						
Ground-glass opacities	59 (66.3)	11 (64.7)	8 (61.5)	40 (67.8)	0.813	0.894
Local pneumonia	14 (15.7)	4 (23.5)	3 (23.1)	7 (11.9)	0.218	0.341
Bilateral pneumonia	75 (84.3)	13 (76.5)	10 (76.9)	52 (88.1)	0.218	0.341
Interstitial abnormalities	5 (5.6)	0 (0)	1 (7.7)	4 (6.8)	0.659	0.641
*Treatment*						
Oxygen uptake (n, %)^c^					0.272	0.159
Without oxygen uptake	21 (23.3)	2 (11.8)	3 (21.4)	16 (27.1)		
Oxygen inhalation through nasal/mask oxygen inhalation (2–7L/min)	66 (73.3)	15 (88.2)	9 (64.3)	42 (71.2)		
NIPPV & IMV	3 (3.3)	0 (0)	2 (14.3)	1 (1.7)		
Antibiotic use (n, %)^c^					0.263	0.464
Coadministration	53 (58.9)	12 (70.6)	9 (64.3)	32 (54.2)		
Without coadministration	37 (41.1)	5 (29.4)	5 (35.7)	27 (45.8)		
Length of stay (d) [M (Q1, Q3)]^d^	22.15 (7, 29)	24.67 (15, 28)	21.08 (13, 27)	22.12 (7, 29)	0.451	0.074
Clinical outcome (n, %)^c^					1.000	1.000
Discharged	78 (86.7)	15 (88.2)	12 (85.7)	51 (86.4)		
Stay in hospital	12 (13.3)	2 (11.8)	2 (14.3)	8 (13.6)		

a Hypertension COVID-19 patients compared to nonhypertension COVID-19 patients.

b The multiple comparisons of well-controlled hypertension COVID-19 patients, poorly controlled hypertension COVID-19 patients, and nonhypertension patients.

c Statistical analysis performed with Fisher’s exact probability test.

d Statistical analysis by nonparametric Mann–Whitney U test or Kruskal–Wallis test.

f Statistical difference between the well-controlled and nonhypertension groups (*p* < 0.05).

e Statistical difference between the poorly controlled and nonhypertension groups (*p* < 0.05).

CHD: coronary heart disease; HF: heart failure; AF: atrial fibrillation; NIPPV: noninvasive positive pressure ventilation; IMV: intermittent mandatory ventilation; AST: aminotransferase; CK: creatine kinase; CK-MB: MB isoenzyme of creatine kinase; LDH: lactate dehydrogenase; Cr: creatinine; BUN: blood urea nitrogen; ALT: alanine aminotransferase; TG: triglyceride; LDL-C: low-density lipoprotein cholesterol; HDL-C: high-density lipoprotein cholesterol; CD4: cluster of differentiation 4; CD8: cluster of differentiation 8; IL-6: interleukin-6; CRP: C-reactive protein; PCT: procalcitonin; APTT: activated partial thromboplastin time; PT: prothrombin time.

There was no statistical difference in sex ratio and age between the well-controlled, poorly controlled, and nonhypertensive groups. The most documented comorbidity among the three groups was diabetes. The well-controlled hypertension group had a significantly higher prevalence of coronary disease, heart failure, and diabetes mellitus than the other two groups. The comparison between well-controlled hypertension and nonhypertension cases showed a statistically significant difference in the prevalence of coronary heart disease (35.3 vs 3.4%) and diabetes mellitus (41.2 vs 11.9%). There was a statistically significant difference in the prevalence of heart failure between patients with poorly controlled hypertension and those without hypertension (14.3 vs 0%). The ratio of severe COVID-19 in the well-controlled hypertension group was significantly higher than that in the nonhypertension group (Table 1).

There was no significant difference in the constituent sex ratio and average age among the ACEI/ARB, non-ACEI/ARB, and nonhypertensive groups. However, the comorbidities of coronary heart disease (28 vs 3.4%, *p* = 0.004) were significantly higher in the non-ACEI/ARB group. And heart failure (16.7 vs 0%, *p* = 0.041) was significantly higher in the ACEI/ARB group. In addition, there was also a statistically higher prevalence of coronary heart disease (35.3 vs 3.4%) and heart failure (16.7 vs 0%) in the ACEI/ARB group than in the nonhypertensive group. In terms of signs and symptoms, there was a statistically significant difference in shivering between the ACEI/ARB and nonhypertensive groups (33.3 vs 0%). The ratio of severe COVID-19 in the non-ACEI/ARB group was significantly higher than that in the nonhypertensive group (Table 2).

**TABLE 2:** The comparison of demographic and clinical characteristics, laboratory and radiology findings, clinical measures, clinical outcomes, and severity of COVID-19 among ACEI/ARB, non-ACEI/ARB, and nonhypertension groups.

	Hypertension (n = 31)		Nonhypertension (n = 59)	*P*
	ACEI/ARB (n = 6)	Non-ACEI/ARB (n = 25)		
*Sex (n, %)* ^a^				
Female	3 (50)	13 (52)	33 (55.9)	0.941
Age (years) [M (Q1, Q3)]^b^	66.00 (55.75, 83.00)	63.00 (55.00, 73.00)	64.00 (54.00, 72.00)	0.827
*Clinical characteristics*				
Clinical classification (n, %)^a^				0.002
Severe	3 (50.0)	12 (48.0)_α_	9 (15.3)	
Nonsevere	3 (50.0)	13 (52.0)_α_	50 (84.7)	
*Preadmission comorbidities (n, %)* ^a^				
CHD	1 (16.7)	7 (28)_α_	2 (3.4)	0.004
HF	1 (16.7)	1 (4)	0 (0)_β_	0.041
AF	0 (0)	1 (4.0)	0 (0)	0.344
Diabetes	2 (33.3)	8 (32.0)	7 (11.9)	0.048
Respiratory disease	0 (0)	2 (8.0)	3 (5.1)	0.741
Cancer	0 (0)	2 (8.0)	6 (10.2)	1.000
*Signs and symptoms (n, %)* ^a^				
Fever	6 (100)	16 (64.0)	45 (76.3)	0.186
Cough	3 (50)	12 (48.0)	28 (47.5)	1.000
Sore throat	0 (0)	2 (8.0)	6 (10.2)	1.000
Expectoration	1 (16.7)	6 (24.0)	9 (15.3)	0.601
Shivering	2 (33.3)^f^	0 (0)	8 (13.6)	0.032
Headache	0 (0)	1 (4.0)	4 (6.8)	1.000
Runny nose	1 (16.7)	0 (0)	3 (5.1)	0.202
Diarrhea	0 (0)	3 (12.0)	10 (16.9)	0.695
Muscular soreness	0 (0)	3 (12.0)	5 (8.5)	0.826
Feeble	1 (16.7)	4 (16.0)	13 (22.0)	0.906
Chest tightness	1 (16.7)	9 (36.0)	16 (27.1)	0.633
Anhelation	0 (0)	3 (12.0)	9 (15.3)	0.888
Temperature (°C) [M (Q1, Q3)]	37.80 (36.60, 39.00)	37.99 (36.20, 39.00)	38.10 (36.60, 39.60)	0.806
*Average blood pressure (mmHg) [M (Q1, Q3)]* ^b^				
Systolic blood pressure	131.00 (116.00, 132.00)	132.50 (126.00, 138.00)_α_	121.00 (113.25, 126.00)	0.000
Diastolic blood pressure	74.00 (72.50, 81.00)	79.00 (74.00, 83.25)	75.50 (71.00, 79.00)	0.187
*Laboratory findings*				
Blood routine [M (Q1, Q3)]^b^				
White cell count, ×10^9^/L	5.19 (4.82, 6.40)	5.80 (4.21, 6.76)	4.96 (4.24, 5.79)	0.200
Neutrophil count, ×10^9^/L	3.25 (3.04, 4.60)	3.55 (2.47, 4.50)	3.07 (2.45, 4.07)	0.334
Lymphocyte count, ×10^9^/L	1.41 (0.94, 1.71)	1.31 (0.99, 1.80)	1.22 (0.84, 1.50)	0.380
Hemoglobin, g/L	119.50 (118.00, 139.25)	117.00 (111.00,128.50)	119.00 (111.00, 126.00)	0.439
Platelet count, ×10^9^/L	182.50 (167.25, 240.75)	261.00 (197.50, 327.00)	208.00 (183.00,288.00)	0.150
HCT (%)	35.90 (35.25, 41.30)	35.40 (32.85, 38.60)	34.40 (33.00, 36.90)	0.152
*Cardiac markers [M (Q1, Q3)]* ^b^				
hs-cTnI (ng/L)	6.60 (1.95, 53.00)	4.20 (1.75, 11.00)	2.60 (1.30, 5.60)	0.144
AST (U/L)	28.00 (20.50, 45.00)	37.50 (24.50, 45.25)	23.00 (19.00, 31.00)	0.044
CK (U/L)	68.00 (50.50, 1,048.25)	73.00 (46.00, 93.50)	64.00 (51.00, 88.50)	0.753
CK-MB (U/L)	1.00 (0.40, 76.75)	0.80 (0.50, 1.40)	0.70 (0.40, 7.85)	0.838
LDH(U/L)	282.00 (158.75, 380.00)	196.00 (162.00, 240.00)	183.00 (150.00, 243.00)	0.328
*Renal and liver function [M (Q1, Q3)]* ^b^				
Cr (μmol/L)	89.50 (65.25, 104.50)	77.00 (63.00, 91.00)	68.90 (63.00, 76.00)	0.137
BUN (mmol/L)	4.45 (2.90, 6.95)	4.60 (4.10, 6.30)	4.40 (3.40, 5.20)	0.383
ALT (U/L)	32.00 (16.75, 48.50)	30.00 (16.00, 50.00)	23.00 (17.00, 35.00)	0.437
*Blood lipid level [M (Q1, Q3)]* ^b^				
TG (mmol/L)	1.24 (0.90, 1.76)	1.56 (0.99, 2.10)	1.26 (0.98, 1.84)	0.695
LDL-C (mmol/L)	1.70 (1.22, 2.12)^f^	2.44 (1.91, 2.77)	2.22 (1.90, 2.48)	0.020
HDL-C (mmol/L)	1.07 (1.00, 1.44)	1.30 (0.1.11, 1.55)	1.20 (1.02, 1.49)	0.457
*Lymphocyte subsets [M (Q1, Q3)]* ^b^				
CD4 (%)	48.14 (33.70, 57.00)	47.14 (40.97, 51.93)	43.34 (37.15, 51.00)	0.508
CD8 (%)	17.62 (11.24, 20.62)	24.03 (19.53, 27.13)	24.08 (18.11, 30.65)	0.100
*Inflammatory markers [M (Q1, Q3)]* ^b^				
IL-6 (pg/ml)	16.90 (6.44, 16.90)	9.61 (6.43, 23.05)	8.97 (5.00, 33.94)	0.819
CRP (mg/L)	1.69 (0.33, 61.06)	5.10 (2.73, 23.13)	2.95 (0.78, 23.98)	0.487
PCT (ng/L)	0.08 (0.06, 0.08)	0.09 (0.05, 0.24)	0.07 (0.04, 0.16)	0.450
*Coagulation test [M (Q1, Q3)]* ^b^				
APTT (sec)	38.55 (35.65, 45.98)	37.20 (35.80, 40.30)	35.90 (33.70, 39.30)	0.088
PT (sec)	12.95 (12.60, 13.70)	13.40 (12.80, 13.95)	13.60 (13.00, 14.10)	0.380
D-dimer (mg/L)	1.05 (0.30, 2.27)	0.68 (0.40, 1.30)	0.47 (0.20, 1.14)	0.339
*Arterial blood gas [M (Q1, Q3)]* ^b^				
PH	7.41 (7.37, 7.50)	7.41 (7.40, 7.43)	7.41 (7.40, 7.43)	0.983
Lactic acid (mmol/L)	2.00 (1.83, 2.18)	2.10 (1.80, 2.50)	1.80 (1.28, 2.08)	0.141
*Radiology findings*				
Abnormalities on chest CT (n, %)^a^				
Ground-glass opacities	2 (40)	17 (68.0)	40 (67.8)	0.506
Local pneumonia	2 (40)	5 (20.0)	7 (11.9)	0.159
Bilateral pneumonia	3 (60.0)	20 (80.0)	52 (88.1)	0.159
Interstitial abnormalities	0 (0)	1 (4.0)	4 (6.8)	1.000
*Treatment*				
Oxygen uptake (n, %)^a^				0.017
Without oxygen uptake	3 (50.0)^f^	2 (8.0)	16 (27.1)	
Oxygen inhalation through nasal/mask oxygen inhalation (2–7L/min)	2 (33.3)^f^	22 (80.0)	42 (71.2)	
NIPPV & IMV	1 (16.7)	1 (4.0)	1 (1.7)	
Antibiotic use (n, %)^a^				0.532
Coadministration	4 (66.7)	17 (68.0)	32 (54.2)	
Without coadministration	2 (33.3)	8 (32.0)	27 (45.8)	
Length of stay (d) [M (Q1, Q3)]^b^	25.40 (19, 28)	22.55 (13, 28)	22.12 (7, 29)	0.499
Clinical outcome (n, %)^a^				1.000
Discharged	5 (83.3)	22 (88.0)	51 (86.4)	
Stay in hospital	1 (16.7)	3 (12.0)	8 (13.6)	

a Statistical analysis performed with Fisher’s exact probability test.

b Statistical analysis by nonparametric Mann–Whitney U test or Kruskal–Wallis test.

c The multiple comparisons of using ACEI/ARB hypertension COVID-19 patients, non-ACEI/ARB hypertension COVID-19 patients, and nonhypertension patients.

d Statistical difference between the non-ACEI/ARB and nonhypertension groups (*p* < 0.05).

e Statistical difference between the ACEI/ARB and nonhypertension groups (*p* < 0.05).

f Statistical difference between the ACEI/ARB and non-ACEI/ARB groups (*p* < 0.05).

CHD: coronary heart disease; HF: heart failure; AF: atrial fibrillation; NIPPV: non-invasive positive pressure ventilation; IMV: intermittent mandatory ventilation. ACEI: angiotensin-converting enzyme inhibitors; ARB: angiotensin receptor blocker. AST: aminotransferase; CK: creatine kinase; CK-MB: MB isoenzyme of creatine kinase; LDH: lactate dehydrogenase; Cr: creatinine; BUN: blood urea nitrogen; ALT: alanine aminotransferase; TG: triglyceride; LDL-C: low-density lipoprotein cholesterol; HDL-C: high-density lipoprotein cholesterol; CD4: cluster of differentiation 4; CD8: cluster of differentiation 8; IL-6: interleukin-6; CRP: C-reactive protein; PCT: procalcitonin; APTT: activated partial thromboplastin time; PT: prothrombin time.

### Antihypertensive Medication History of Patients With Hypertension

All patients with hypertension were documented using several kinds of antihypertensive drugs. Among patients using ACEI/ARB agents before hospitalization, 3 (50%) used ACEI agents, whereas the other 3 (50%) used ARB agents. Among those not using ACEI/ARB agents, 14 (56%) patients used calcium channel blockers, 5 (20%) used β-receptor blockers, 1 (4%) used adrenoceptor blocking agents, and 3 (12%) used diuretics. In addition, 6 (24%) patients did not use antihypertensive drugs before hospitalization. Moreover, 30 patients received antihypertensive treatment after admission.

The average blood pressure during hospitalization was recorded for each group. The average blood pressure was found to be statistically different between the hypertension and nonhypertension groups. The average blood pressure during hospitalization showed no statistical difference between well-controlled and poorly controlled groups. Similarly, there was also no statistical difference in the average blood pressure during hospitalization for comparison between ACEI/ARB and non-ACEI/ARB groups (Tables 1 and 2).

### Laboratory and Radiological Findings

The level of aspartate aminotransferase (AST) was significantly higher in the hypertensive group than in the nonhypertensive group (*p* = 0.018). And there was borderline significance in the comparison of hs-cTnI levels between the hypertension and nonhypertension control groups (*p* = 0.057). For the coagulation test and arterial blood gas, activated partial thromboplastin time (APTT) and lactic acid levels were substantially lower in the nonhypertension group than in the hypertension group. In comparison among the three subgroups of well-controlled, poorly controlled, and nonhypertensive groups, the results showed a statistically significant difference in AST levels (Table 1).

**FIGURE 2:**
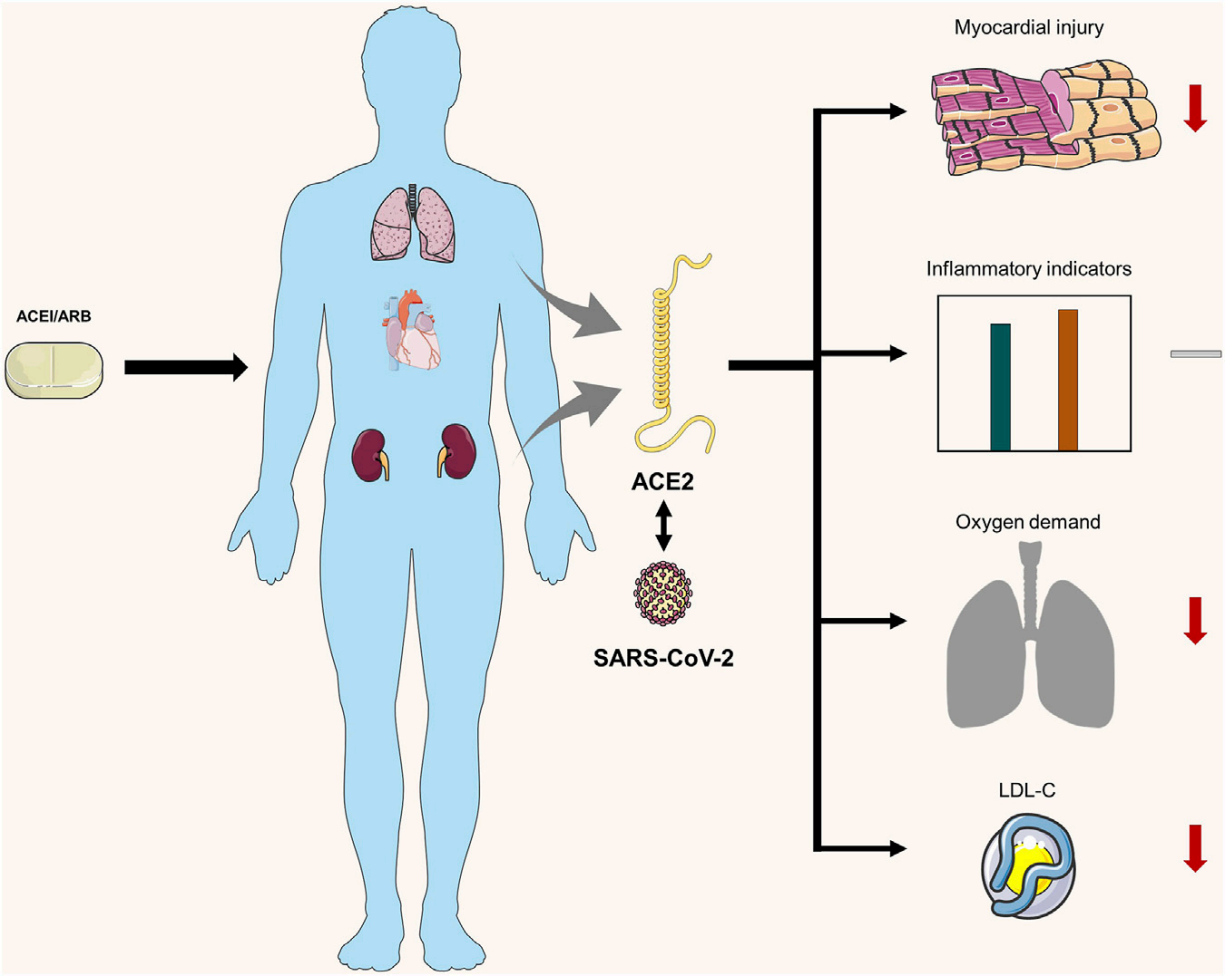
Summary findings of the impact of ACEI/ARB agents on COVID-19 patients in our study.

For cardiac markers and blood lipid levels, AST showed statistically significant differences among the three groups, ACEI/ARB, non-ACEI/ARB, and nonhypertensive groups (28.00U/L vs 37.50U/L vs 23.00U/L, respectively; *p* = 0.044). The level of LDL-C was significantly higher in the non-ACEI/ARB group than in the ACEI/ARB group (2.44 mmol/L vs 1.70 mmol/L) (Table 2 and Figure 2).

### Lymphocyte Subsets and Inflammatory Markers

The blood routine, lymphocyte subsets, and inflammatory markers in the three groups of patients were compared. The results of the comparison between the ACEI/ARB, non-ACEI/ARB, and nonhypertensive groups showed no statistical differences in white cell count (5.19/L vs 5.80/L vs 4.96 × 10^9^/L, respectively; *p* = 0.200), neutrophil count (3.25/L vs 3.55/L vs 3.07 × 10^9^/L, respectively; *p* = 0.334), and lymphocyte count (1.41/L vs 1.31/L vs 1.22 × 10^9^/L, respectively; *p* = 0.380) (Table 2 and Figure 2).

Similarly, the comparison for lymphocyte subsets and inflammatory factors between the ACEI/ARB, non-ACEI/ARB, and nonhypertensive groups showed no statistical differences in CD4 (48.14 vs 47.14% vs 43.34%, respectively; *p* = 0.508), CD8 (17.62 vs 24.03% vs 24.08%, respectively; *p* = 0.100), IL-6 (16.90 pg/ml vs 9.61 pg/ml vs 8.97 pg/ml, respectively; *p* = 0.819), C-reactive protein (1.69 mg/L vs 5.10 mg/L vs 2.95 mg/L, respectively; *p* = 0.487), and procalcitonin (0.08 ng/L vs 0.09 ng/L vs 0.07 ng/L, respectively; *p* = 0.450) (Table 2 and Figure 2).

### Treatment and Clinical Outcomes

Of the 90 patients, 78 were discharged and 12 were transferred to another hospital because of ward closure. The clinical outcomes of COVID-19 patients were as follows: 27 (87.1%) hypertension cases and 51 (86.4%) nonhypertension cases were cured and discharged, and 4 (12.9%) hypertension cases and 8 (13.6%) nonhypertension cases remained in the hospital, including 2 (3.2%) patients requiring intubation and mechanical ventilation. Furthermore, there were no significant differences in oxygen uptake, length of stay, and clinical outcomes among the groups (Table 1).

The comparison of differences in oxygen uptake among the three groups showed statistically significant results (*p* = 0.017). The nonoxygen uptake cases in the ACEI/ARB group were significantly higher in the pairwise comparison than in the non-ACEI/ARB group (Table 2 and Figure 2).

### Severity of COVID-19-Related Factors

Risk factors were identified through univariate logistic regression, which included sex, age, uncontrolled hypertension, and antihypertensive medication history. The results revealed that compared with patients without hypertension, poorly controlled (OR = 3.97, 95% CI = 1.03–15.30, *p* = 0.045) or well-controlled (OR = 6.48, 95% CI = 1.77–23.81, *p* = 0.005) hypertension patients who did not use ACEI/ARB antihypertensive agents had a higher risk of developing severe COVID-19. A multivariate logistic regression analysis showed similar findings (poorly controlled hypertension patients without using ACEI/ARB: OR = 4.03, 95% CI = 1.01–16.07; well-controlled hypertension patients without using ACEI/ARB: OR = 6.09, 95% CI = 1.77–26.48) (Table 3).

**TABLE 3:** Univariate logistic regression analysis and multivariate logistic regression analysis of severity of COVID-19-related factors.

Items	Univariate			Multivariate	
	OR (95% CI)	*P*	OR (95% CI)		*P*
Sex					
Male	1.000		1.000		
Female	0.50 (0.19–1.28)	0.146	0.46 (0.16–1.34)		0.156
Age	1.03 (0.99–1.06)	0.196	1.02 (0.985–1.07)		0.234
Hypertension					
Nonhypertension	1.000		1.000		
Well-controlled and using ACEI and ARB	5.56 (0.69–44.67)	0.107	4.48 (0.53–38.07)		0.170
Poorly controlled and using ACEI/ARB	5.56 (0.32–97.13)	0.240	7.01 (0.37–138.08)		0.193
Well-controlled and not using ACEI/ARB	6.48 (1.7–23.81)	0.005	6.85 (1.77–26.48)		0.005
Poorly controlled and not using ACEI/ARB	3.97 (1.03–15.30)	0.045	4.03 (1.01–16.07)		0.048

ACEI: angiotensin-converting enzyme inhibitors; ARB: angiotensin receptor blocker; OR: odds ratio; CI: confidence interval.

## Discussion

This retrospective cohort study initially revealed the clinical characteristics and inflammatory immune responses of COVID-19 patients with and without hypertension. In this study, patients with hypertension were more likely to have increased oxygen demand, myocardial injury, and greater possibilities of developing severe COVID-19, suggesting that hypertension might play an important role in COVID-19. These findings are consistent with the idea that hypertension cases have an increased risk of comorbidity, infection, and multiple organ function damage (Volpe et al., 2015; Qaseem et al., 2017; Guo et al., 2020; Huang et al., 2020). Not only that, in the analysis of different subgroups, the result also showed a difference in the level of AST, LDL-C, and demand for oxygen among subgroups. However, no significant differences in lymphocyte subsets and inflammatory markers were found between the subgroups. These findings are in line with previous studies (Bansal, 2020; Kreutz et al., 2020; Lopes et al., 2021).

Hypertension is an important comorbidity in patients with COVID-19. The immune activation in hypertension patients that was largely augmented under COVID-19 raises a concern about the negative effect of hypertension COVID-19 patients (Trump et al., 2021). In our study, levels of aminotransferase (AST) and hs-cTnI were high in the hypertension group. Although the relationship between myocardial injury and COVID-19 with hypertension is underreported to date, hs-cTnI was to be a gold standard biomarker for diagnosing myocardial injury (Nigam, 2007). So it was theorized that COVID-19 patients with hypertension were more likely to develop myocardial injury. Moreover, people with hypertension also had a higher risk of developing the severity of COVID-19. Considering the impact of hypertension on COVID-19 patients, more attention should be paid to COVID-19 patients with hypertension in clinical practice. In addition, clinicians should pay more attention to the cardiac damage of COVID-19 patients with hypertension and early interventions are warranted.

The RAAS plays a major role in the pathophysiology of hypertension (Lu et al., 2015). SARS-CoV-2 uses ACE2 as a viral entry receptor, (Bosso et al., 2020), although there is no direct evidence for whether the use of RAAS inhibitors is beneficial to the treatment or prognosis of COVID-19. Animal experiments also showed that ACEI/ARBs treatment would increase the ACE2 expression (Trump et al., 2021). It was theorized that these medications could enhance viral binding and cell entry (Esler and Esler, 2020). But in our study, the levels of AST and hs-cTnI were high in the hypertension group, and the long-term use of ACEI/ARB had a statistically significantly lower AST, low-density lipoprotein cholesterol (LDL-C), and oxygen uptake and lower white cell count, neutrophil count, and levels of CD4, CD8, CRP, and PCT but without statistical significance. Contrary to the above theory, the long-term use of ACEI/ARB agents in patients with hypertension might also provide more protection to the lungs and other organs than the nonuse of ACEI/ARB. In addition, the long-term use of ACEI/ARB agents may suppress the inflammatory response in COVID-19 patients. SARS-CoV-2 is driven by ACE2 downregulation, leading to an array of complex and intertwined molecular interactions via at least four axes consisting of the dysregulation of the ACE2/angiotensin II/AT1R axis, attenuation of ACE2/MasR axis, increased activation of ACE2/bradykinin B1R/DABK axis, and activation of the complement cascades, resulting in a tornado of inflammatory cytokine responses (Tisoncik et al., 2012). However, RAAS inhibitors could benefit patients with COVID-19 through their effects on the angiotensin II expression and subsequent increases in Ang-(1–7) and (1–9), which have vasodilatory and anti-inflammatory effects that might attenuate lung injury, myocardial injury, and injury to other organs (Ingelfinger, 2009; Fraga-Silva et al., 2012; Verdecchia et al., 2012; Santos et al., 2013; Arendse et al., 2019). Although the role of RAAS blockers in patients with COVID-19 has not completely been determined, scientific societies have recommended that patients should not discontinue ACEI or ARB therapy during the COVID-19 pandemic (Celutkiene et al., 2020). The results of our study also support this view. Our clinical findings not only provided evidence on the impact of hypertension on COVID-19 but also provided new insights into the benefits of antihypertensive medication on COVID-19.

This study has several limitations. First, it was a retrospective study, and recall bias on testing or diagnosis may occur in some patients. Second, the relatively small number of patients with hypertension who are taking ACEI/ARBs might limit the extension of these results to a broader population. Third, the effect of ACEIs or ARBs on the susceptibility to COVID-19 was not studied because the focus was only on clinical outcomes in patients already been infected. Fourth, the duration and doses of the antihypertensive medication use were not systematically collected.

## Conclusion

In conclusion, hypertension, especially poorly controlled hypertension, may play an important role in the severity of COVID-19. COVID-19 patients with hypertension are more likely to develop myocardial injury. The long-term use of ACEI/ARB agents might also provide more protection to the lungs and other organs, as well as suppress the inflammatory response in COVID-19 patients with hypertension. These findings suggest that the use of renin–angiotensin system blockers may be beneficial for COVID-19 patients with hypertension.

## Data Availability

The raw data supporting the conclusions of this article will be made available by the authors, without undue reservation.
